# More evidence for widespread antagonistic pleiotropy in polymorphic disease alleles

**DOI:** 10.3389/fgene.2024.1404516

**Published:** 2024-06-17

**Authors:** Cynthia Lockwood, Ashley S. Vo, Hanna Bellafard, Ashley J. R. Carter

**Affiliations:** California State University Long Beach, Department of Biological Sciences, Long Beach, CA, United States

**Keywords:** antagonistic pleiotropy, genetic disease, balancing selection, heterozygote advantage, overdominance

## Abstract

**Introduction:**

Many loci segregate alleles classified as “genetic diseases” due to their deleterious effects on health. However, some disease alleles have been reported to show beneficial effects under certain conditions or in certain populations. The beneficial effects of these antagonistically pleiotropic alleles may explain their continued prevalence, but the degree to which antagonistic pleiotropy is common or rare is unresolved. We surveyed the medical literature to identify examples of antagonistic pleiotropy to help determine whether antagonistic pleiotropy appears to be rare or common.

**Results:**

We identified ten examples of loci with polymorphisms for which the presence of antagonistic pleiotropy is well supported by detailed genetic or epidemiological information in humans. One additional locus was identified for which the supporting evidence comes from animal studies. These examples complement over 20 others reported in other reviews.

**Discussion:**

The existence of more than 30 identified antagonistically pleiotropic human disease alleles suggests that this phenomenon may be widespread. This poses important implications for both our understanding of human evolutionary genetics and our approaches to clinical treatment and disease prevention, especially therapies based on genetic modification.

## Introduction

Mutations in wildtype alleles can have deleterious effects on individuals and their persistence leads to polymorphic genetic disorders. Why genetic disorders persist when natural selection is an effective mechanism to favor advantageous alleles and eliminate deleterious ones is a complex question. Possible mechanisms include genetic drift or mutation selection balance, but if a mutation in an allele causes a deleterious effect while also improving some other trait, such antagonistic pleiotropy can lead to the long-term persistence of deleterious alleles by natural selection (Carter and Nguyen, 2011; Withrock et al., 2015; Ukraintseva et al., 2016; Byars and Voskarides, 2020).

Antagonistic pleiotropy has historically been mainly considered in the context of how organisms acquire their wildtype alleles. Traits that manifest during peak reproductive ages have the strongest influence on overall fitness whereas traits selected after the peak reproductive period are more weakly selected. Antagonistically pleiotropic mutations that benefit younger individuals while harming older ones may therefore have an overall positive effect and a history of the fixation of mutations with these age-specific benefits and detriments is widely thought to explain the presence of senescence (Medawar, 1951; Williams, 1957; Kirkwood and Rose, 1991).

What has been less commonly considered is how antagonistic pleiotropy may contribute to genetic variation within populations. Studies of genetic variation in this context are more recent than the first set of classic paper described above. Mutations classified as disease alleles may result in higher overall fitness than expected if they also provide unrecognized offsetting benefits. Natural selection can also actively maintain multiple alleles that are individually deleterious if they provide benefits in different individuals, under different environmental conditions, or act together to increase fitness in heterozygotes (i.e., overdominance) (Bitarello et al., 2023).

Environmental and historical context is important; the advantages that cause high frequencies in modern populations may or may not still be present today. For example, Crohn’s disease may be so common today because of the bubonic plague (*Y. pestis*) in Medieval Europe. An allele modifying the ERAP2 transcript shows evidence of positive selection during the plague and data shows that this protein influences cytokine response to *Yersinia pestis* exposure (Klunk et al., 2022), but this allele is a risk factor for Crohn’s disease in modern populations (Di Narzo et al., 2016). A better-known example of this kind of historically influenced antagonistic pleiotropy is sickle cell disease in which homozygotes for the Hb-S allele have poor life expectancies due to blood cell malformations, but heterozygotes for the Hb-S allele have increased resistance to malaria during early childhood (Aidoo et al., 2002). The historical advantage explains the relatively high frequency of the allele in Africa, tropical, and Mediterranean regions where the historical and modern risks of malaria are high (Ashley-Koch et al., 2000).

These two examples illustrate that antagonistic pleiotropy may be important for the prevalence of Crohn’s disease and sickle cell disease, but how widespread is this phenomenon? To what extent is the overall prevalence of genetic disease due to antagonistic pleiotropy? Despite the potential importance of this process, the idea has received little attention in medical literature (Leroi et al., 2005; Key et al., 2014).

Therefore, following the approach of several previous authors (Carter and Nguyen, 2011; Withrock et al., 2015; Ukraintseva et al., 2016; Byars and Voskarides, 2020) we performed a search of the literature to identify and describe examples of alleles associated with increased risk of human disease which also confer a health or fitness benefit. As the number of identified examples of such alleles increases, the importance of antagonistic pleiotropy as an explanation for the prevalence of human genetic disease becomes likely.

Searches were conducted using PubMed and Web of Science using terms such as " antagonistic pleiotropy”, “balancing selection”, “overdominance” and “heterosis” and abstracts were screened for further reading. We did not specify a limited set of journals or time frame, so this analysis represents an exploration rather than a formal meta-analysis. To avoid duplication of the information described previously (Carter and Nguyen, 2011; Withrock et al., 2015; Ukraintseva et al., 2016; Byars and Voskarides, 2020) we omit providing detailed descriptions for the diseases identified in those studies unless we found meaningful additional evidence not described therein. The goal of this article is not to be exhaustive in scope or detail, but rather to highlight well-supported examples of antagonistic pleiotropy and contribute to an appreciation of their prevalence.

## Results

### A-kinase-anchoring protein 2 polymorphism

The dual-specificity A-kinase-anchoring protein 2 (D-AKAP2, encoded by the AKAP10 gene) binds protein kinase A (PKA) and is important for the subcellular localization and functionality of PKA, a broad serine/threonine protein kinase that regulates a variety of cellular processes including early development (Huang et al., 1997; Paolillo et al., 2022). In humans, the AKAP10 locus has two common alleles defined by an A/G SNP at position 1936 which cause an isoleucine/valine polymorphism at position 646 of the D-AKAP10 protein. Carriers of the G allele have been reported to exhibit an increased risk of breast (Wirtenberger et al., 2007) and colorectal cancers (Wang et al., 2009), increased basal heart rate (Tingley et al., 2007), increased risk of myocardial infarction (Nishihama et al., 2007; Yoshida et al., 2007), and decreased heart rate variability (Neumann et al., 2009).

Presence of the G allele at the AKAP10 locus is also associated with decreased preterm birth, however. A study of 203 newborns showed that preterm newborns were 55% more likely to be AA homozygotes than full term newborns, indicating a positive association between the G allele and full-term birth (Łoniewska et al., 2012). Those authors suggested that newborns homozygous for the G allele may be more efficient at changing from glycolytic to oxidative metabolism after birth.

### Angiotensin-converting enzyme polymorphism

Angiotensin-converting enzyme (ACE) cleaves angiotensin I into angiotensin II which stimulates aldosterone synthesis and leads to blood vessel constriction (Studdy et al., 1983). In humans, the ACE locus has a highly-studied polymorphism defined by the insertion *I*) or deletion *D*) of 287 base pairs in intron 16, with the presence of the D allele raising the levels of ACE protein in plasma (Rigat et al., 1990). Increased levels of ACE protein and the *D* allele have been associated with an increased risk of hypertension (Higaki et al., 2000; Montes-de-Oca-García et al., 2021), coronary artery disease (Nakai et al., 1994), myocardial infarction (Chen et al., 2013), polycystic ovary syndrome (Ożegowska et al., 2016), and prostate cancer (Du et al., 2022).

Possession of the D allele appears to improve athletic performance in power and sprint exercises however (Maciejewska-Skrendo et al., 2019); higher ACE levels appearing to provide benefits in some acute cardiovascular situations at the expense of the chronic detrimental effects described above. The *D* allele may also provide protection from Alzheimer’s disease. A meta-analysis of 39 studies indicated that *DD* homozygotes displayed a significantly reduced risk of Alzheimer’s disease (Lehmann et al., 2005). Even though Alzheimer’s disease is typically observed in individuals of post reproductive age, reduced risk of this disease may be selectively beneficial due to the “grandmother effect” whereby individuals provide care and resources to their grandchildren and improve their survival, a form of improved overall reproductive fitness (Lachmann, 2011). In addition, a case-control study with 440 subjects suggested that males homozygous for the *D* allele experience reduced risk of migraines (Lin et al., 2005). Finally, the D allele may also be protective against infectious diseases like SARS-CoV-2 (Delanghe et al., 2020), but the evidence is mixed (e.g., Sousa et al., 2023).

### Apolipoprotein E polymorphism

Apolipoprotein E (ApoE) plays a role in cholesterol metabolism by transporting cholesterol and other fats through the blood (Yang et al., 2023). In humans, the ApoE locus has three commonly occurring alleles defined by the amino acids present at positions 112 and 158 (apoE4 Arg112/Arg158, ApoE3 Cys112/Arg158, and ApoE2 Cys112/Cys158) which are referred to as the E4, E3 and E2 alleles respectively. The E4 allele appears to be ancestral with E3 arising later and E2 arising from the E3 allele (McIntosh et al., 2012). Carriers of the E4 allele have greater cholesterol levels than carriers of the E3 and E2 alleles, possibly due to more efficient intestinal absorption (Tikkanen et al., 1990), and possession of the E4 allele is associated with increased risk of hypertension (Niu et al., 2009), cardiovascular disease (Song et al., 2004) and Alzheimer’s disease (Goldman, 2012; O'Neil, 2023; Liampas et al., 2024).

In menstruating women, those with at least one E4 allele displayed 20% higher levels of luteal progesterone, a hormone vital for maintaining the endometrium during early pregnancy and important for a successful pregnancy, compared to women without the E4 allele (Jasienska et al., 2015). Consistent with this, the E4 allele was associated with 12% and 30% increases in fecundity (for one or two E4 copies respectively compared to the E3 allele) in a population of forager-horticulturalists (Trumble et al., 2023). Other benefits provided by the E4 allele include increased cognitive functions in young adults (Wozniak et al., 2002) resistance to certain liver diseases caused by hepatitis C virus (Wozniak et al., 2002), improved cardiac performance (Topriceanu et al., 2024), better visual working memory (Lu et al., 2021), protection against diarrhea in children during their first 2 years of life (Oriá et al., 2005), and protection from negative effects on linguistic and categorical tasks in children who did suffer severe diarrhea (Oriá et al., 2005).

### BRCA1 and BRCA2 tumor suppressor polymorphisms

BRCA1 and BRCA2 are tumor suppressor genes that are responsible for DNA repair, regulation of cell division, and maintenance of chromosomal stability (Scully and Livingston, 2000). There are several polymorphisms of BRCA1 and BRCA2 associated with increased risk of sex-specific cancers such as breast cancer (Valentini et al., 2024), ovarian cancer (Risch et al., 2001; Horackova et al., 2023), and endometrial cancer (Sorouri et al., 2023)in women and prostate cancer in men (Kalampokis et al., 2024). Both men and women with BRCA2 mutations also appear to have increased risks of gastric cancer (Buckley et al., 2022), pancreatic cancer (McGarry et al., 2022) and melanoma (Toussi et al., 2020). In addition to cancer risk, possession of BRCA mutations was associated with increased non-cancer mortality in a population of over 5,000 subjects (Mai et al., 2009).

Mutations in the BRCA1 and BRCA2 loci are associated with increased reproduction, however. In a pedigree-based study of women living in the early part of the 20th century in Utah, women with these mutations appeared to have approximately two more children than control individuals and were 2.04–3.6 times more likely to have four or more children than individuals from the control population (Smith et al., 2012). A similar study of French Women showed a more modest, but significant, fertility increase in women with mutations compared to family members without (Kwiatkowski et al., 2015). The exact mechanism of this benefit is unknown, but evidence suggests that the BRCA1 mutation decreases shortening of telomeres (Ballal et al., 2009) and longer telomere lengths have been associated with increased reproductive lifespan in women (Aydos et al., 2005).

### Cystic fibrosis transmembrane conductance regulator polymorphism

Cystic fibrosis (CF) is an autosomal recessive disorder caused by various mutations in the cystic fibrosis transmembrane conductance regulator (CFTR) gene, but the ΔF508 mutation (a deletion of the phenylalanine at amino acid position 508 in the protein) accounts for 70% of CF patients (Kerem et al., 1989). Mutations in the CFTR gene encode a nonfunctional chloride channel, causing a broad range of deleterious effects (Ramananda et al., 2024) including obstructive lung disease and increased susceptibility to respiratory infections (Rowntree and Harris, 2003), pancreatic disorders (Estivill et al., 1995), and reduced fertility in both males (Alves et al., 2015) and females (Brunoro et al., 2011).

In contrast, comparisons of families with hereditary CF to control families show increased sizes (Knudson et al., 1967), likely arising from increased reproduction in heterozygous carriers. CFTR mutations may also provide resistance to infectious disease by reducing the amount of sulfate available to *Mycobacterium tuberculosis* and cause lower rates of tuberculosis infection (Tobacman, 2003). Theoretical population genetic modelling of the historical and modern exposures to tuberculosis suggests that resistance conferred by CFTR mutations may account for the modern geographic distribution and prevalence of CFTR mutations (Poolman and Galvani, 2007; Lubinsky, 2012; Bosch et al., 2017). Cystic fibrosis follows a modern geographical distribution that tracks with the historical presence of large urban centers when tuberculosis risk was greatest; the highest incidence occurs in European newborns (1 in 2,500) with lower rates in Indian populations (1 in 40,000) (Powers et al., 1996) and African and East Asian populations (extremely low) (Welsh et al., 2001). The 17th century tuberculosis epidemic in Europe followed by a more recent spread to India and Africa during the 19th century (Bates and Stead, 1993) is consistent with the difference in modern rates of cystic fibrosis. Additionally, mice that are heterozygous for CFTR mutations show resistance to cholera toxin (Gabriel et al., 1994) which suggests a likely similar benefit for humans. A similar protective effect, based on pH and physical properties of mucus, was also reported for SARS-CoV-2 patients (Tedbury et al., 2023), but other studies disagree (Baldassarri et al., 2021).

### Hereditary hemochromatosis polymorphism

Mutations in the hemochromatosis (HFE) gene cause hereditary hemochromatosis (HH), a disorder categorized by excessive accumulation of iron in parenchymal organs. The most common mutation in the HFE gene, seen in up to 1 in 8 individuals in certain European regions, is one in which a cysteine is replaced by tyrosine at position 282, the p.Cys282Tyr allele (Hollerer et al., 2017). This mutation impairs binding to β2-microglobulin and as a result the mutant HFE protein is unable to reach the cell surface and aggregates intracellularly (Hollerer et al., 2017). Symptoms of HH include chronic fatigue, hyperpigmentation, joint and bone symptoms to diabetes, and liver diseases such as fibrosis, cirrhosis, and hepatocellular carcinoma (Hollerer et al., 2017).

Recent studies have shown that the p.Cys282Tyr allele can positively influence the immune system, improve the general fitness and reproductive status of mutation carriers, and reduce the risks of amyotrophic lateral sclerosis, Alzheimer’s disease, Parkinsons disease, and atherosclerosis (Hollerer et al., 2017). Additional evidence for a general benefit to physical fitness comes from data showing that 80% of successful French athletes were heterozygous for one of several HFE mutations (Hermine et al., 2015) and a large-scale study among a Sicilian population showing that p.Cys282Tyr heterozygous individuals, specifically women, tended to have significantly higher life expectancies compared to controls (Balistreri et al., 2002; Lio et al., 2002). This allele may also protect against the effects of *M. tuberculosis* due to reduced iron levels of macrophages in p.Cys282Tyr allele carriers (Olakanmi et al., 2007; Weinberg, 2008).

### Human leukocyte antigen polymorphism

The human leukocyte antigen (HLA) complex exhibits highly polymorphic alleles that are responsible for producing the different haplotypes of the major histocompatibility complex (MHC) (Choo, 2007; Shiina and Kulski, 2024). The wide diversity of HLA alleles is needed for the immune system to function successfully (Bjorkman et al., 1987; Traherne, 2008), but specific alleles have been shown to have deleterious effects with the most highly studied of these being the 8.1 ancestral haplotype (AH). The 8.1 AH is associated with increased risk of non-Hodgkin lymphoma (Wang et al., 2011), colorectal and ovarian cancer (Tóth et al., 2007), diabetes (Price et al., 1999), and autoimmune disorders such as systemic lupus and IgA deficiency (Price et al., 1999) and myositis (Miller et al., 2015).

In contrast, the presence of the 8.1 AH allele is associated with longevity in males as evidenced by nonagenarian males having a higher frequency of the 8.1 AH allele compared to young males and nonagenarian females (Rea and Middleton, 1994; Caruso et al., 2000). The 8.1 AH allele appears to provide protective effects against narcolepsy (Hor et al., 2010). The 8.1 AH allele appears to have modest beneficial effects on human gut microbiota (Hov et al., 2015), and for cystic fibrosis patients it is associated with delayed colonization of *Staphylococcus aureus and Pseudomonas aeruginosa* (Laki et al., 2006; D'Antonio et al., 2019) and reduced risk of septic shock (Aladzsity et al., 2011).

### Machado-Joseph disease polymorphism

Machado-Joseph disease (MJD), also called spinocerebellar ataxia Type 3, is an autosomal dominant neurodegenerative disorder where individuals experience progressive clumsiness, lurching gait, impaired eye movement, and dystonia which lacks effective treatments (Oliveira et al., 2022). Onset of MJD occurs around the age of 30 and is attributed to a high number of CAG trinucleotide repeats (particularly more than 60 repeats) within the MJD1 locus (also called ATXN3) (Paulson, 2012).

Expansion of the CAG repeats in the MJD1 locus and the consequent MJD is associated with increased reproduction, however. An analysis of 82 Brazilian families with MJD found that affected women had 45% more children than the general population and were more likely to have their first child and undergo menopause at a younger age than both the unaffected members of their families and the general population (Prestes et al., 2008; Sena et al., 2021).

### Methylenetetrahydrofolate reductase polymorphism

MTHFR (Methylenetetrahydrofolate reductase), an enzyme involved in the process of adding a methyl group to folic acid to make it useable by the body, has two clinically relevant deleterious polymorphisms (Dean, 2012) which reduce folate levels. At position 677 of MTHFR there is a C/T SNP and individuals heterozygous or homozygous for the T nucleotide produce a MTHFR protein with reduced activity which leads to lower levels of folate and higher levels of homocysteine in the blood. Clinically, in addition to increased risks of a variety of birth defects from the presence of the T allele (Pi et al., 2020), is also associated with higher risks of schizophrenia (Lewis et al., 2005), male infertility (Karimian and Colagar, 2016; Aliakbari et al., 2020), gestational diabetes (Tan and Chen, 2023) and recurrent pregnancy loss (Wu et al., 2012) in Asians, and Alzheimer’s disease in Asians (Hua et al., 2011; Peng et al., 2014) and APOE4 carriers (Peng et al., 2014). At position 1,298 of MTHFR there is an A/C SNP with a similar biochemical effect but where the effects of the C allele are generally less severe, but evidence shows an association with increased cervical cancer risk (Yi et al., 2016) and reduced sperm counts in Asian (Aliakbari et al., 2020), Moroccan (Eloualid et al., 2012), and Indian (Singh et al., 2010) populations. Both alleles have been associated with reduced fertility in Asians (Shi et al., 2019) and increased risk of polycystic ovary syndrome and ovarian cancer (Xiong et al., 2020).

These alleles appear to provide protection against some cancers, however. Two metanalyses indicated that homozygosity at position 677 for the T Allele was associated with a significantly reduced rate of colorectal cancer (Hubner and Houlston, 2006; Huang et al., 2007) and one of them indicated the same for the C allele at position 1,298 (Hubner and Houlston, 2006). A pair of meta-analyses looking at prostate cancer risk reported that the T allele at position 677 was associated with reduced prostate cancer risk in an Asian population (You et al., 2024) whereas the C allele at position 1,298 was associated with reduced prostate cancer risk in a European population (Chen et al., 2015).

### Niemann-Pick C1 disease polymorphism

Niemann-Pick C1 (NPC1) disease is a recessive autosomal disease caused by mutations that decrease NPC1 protein activity and result in an accumulation of LDL cholesterol in lysosomes (Carstea et al., 1997; Lloyd-Evans et al., 2008) which affects roughly 1 in 92,104 (Wassit et al., 2016). NPC1 disease causes liver, lung, spleen, brain, and motor control problems and the afflicted typically die before adulthood (Sarna et al., 2003; Lamri et al., 2018; Wheeler and Silence, 2020). NPC1 disease alleles are also associated with neurological problems in murine models due to Purkinje cell degeneration (Sarna et al., 2003) and NPC1 knockout mice showed a formation of aortic atherosclerosis when fed a high lipid diet, indicating that functional NPC1 likely provides protective effects against atherosclerosis (Zhang et al., 2008).

Increased storage of fat may have been advantageous in historical periods of famine, and genomic evidence suggests that balancing selection favoring heterozygotes may have occurred in the past (Chiorean et al., 2020). Reduced levels of the functional NPC1 protein may also protect against some viral infections. Tissue culture evidence demonstrates that the NPC1 protein is needed for host cell entry by the Ebola (Carette et al., 2011; Côté et al., 2011) and Marburg (Carette et al., 2011) filoviruses for viral entry. An NPC1 knockdown study using human and murine cells infected with the Ebolavirus showed a virus titer reduction of greater than 99% in the infected cells (Sadewasser et al., 2019). NPC1 homozygous knockout mice exposed to Mouse-adapted versions of these viruses were highly resistant whereas control mice mutations died quickly, but heterozygous mice demonstrated increased virus resistance without NPC1 disease problems (Herbert et al., 2015). Consistent with this, studies using cell lines derived from green monkey and human tissue showed a decrease in the ability of Ebola and Marburg viral analogs to infect cells with SNPs inserted into the NPC1 sequence (Kondoh et al., 2018). Finally, several antiviral drugs work by blocking the NPC1 protein in patients (Ahmad et al., 2023), suggesting that heterozygotes are likely to experience increased resistance to such infections.

### RH factor polymorphism

The Rh blood group system consists of two tightly linked loci, a D locus, and a C/E locus (Avent and Reid, 2000) with a polymorphism for a full deletion of the D locus and its encoded D allele protein defining the clinically important Rh positive/negative blood group. While evidence exists for a variety of poorer health metrics in Rh negative individuals than in Rh positive individuals (Flegr et al., 2015), the largest impact on fitness involves reproduction. Pregnant women with Rh-/Rh- genotypes can mount an immune system attack against their Rh+/Rh- fetuses if their bodies have become sensitized to the Rh + antigens (typically by a previous pregnancy). This immune system attack targets the red blood cells of the fetus and can lead to serious anemia and death, with fetal mortality estimated at 24% when untreated (Bhutani et al., 2013). Despite this risk, the Rh- allele is present in individuals of European ancestry at frequencies of approximately 15%, with some populations as high as 40%, while being much rarer in other populations (Perry et al., 2012).

Interestingly, the risk of the Rh- allele also works in a frequency dependent manner. In populations in which the Rh- allele becomes common, its relative detriment declines because Rh + males begin to experience reduced fitness (due to mating with Rh- females) while the cost of being a Rh- female is ameliorated by the higher frequency of Rh+/Rh- and Rh-/Rh- males who pose less risk. Modelling of this situation (Haldane, 1941; Perry et al., 2012) indicates that at the frequencies seen in Europe, the net strength of selection against the Rh- allele is very weak. Consistent with this is a study of 112 Hutterite families (a population with large families and well recorded pedigrees), in which families at risk (i.e., Rh- women with Rh + husbands) had only 3% fewer children on average and this difference was not significant (*p* > 0.7) (Potter, 1948).

On the beneficial side, A study on women in Baltimore also reported that Rh- women of European ancestry had significantly more living children than Rh + ones (Glass, 1950). The Rh negative allele has also been reported to be associated with reduced pregnancy induced hypertension (Dahlén et al., 2021), and appears to provide protection against some infectious diseases such as Chikungunya fever (Lokireddy et al., 2009; Kumar et al., 2010), and SARS-Cov-2 (Zietz et al., 2020; Butler et al., 2023).

## Discussion

We have described 10 loci with alleles where antagonistic pleiotropy was demonstrated in humans and one (NPC1) where evidence from animal studies and modelling suggests it is likely to exist. Table 1 summarizes these results. Our previous paper (Carter and Nguyen, 2011) identified 7 examples strongly supported by data in humans and 7 others with support from animal studies. Other articles have described 6 strong examples (Byars and Voskarides, 2020), 7 strong examples with 5 suggestive ones (Withrock et al., 2015), and 12 suggestive examples (Ukraintseva et al., 2016). Some of these examples overlap, but there is now good evidence for antagonistic pleiotropy being involved in 39 genetic disorders (Table 2).

**TABLE 1:** Antagonistically pleiotropic disease alleles described in this paper. The effects of these polymorphisms are well-supported by detailed human genetic or epidemiological information, except for NPC1 where the evidence is based on animal models. For the alleles listed, the one associated with the disease or deleterious effect is listed first.

Disease/Locus/alleles	Deleterious Effect(s)	Beneficial Effect(s)
A Kinase-Anchoring Protein 2 (AKAP10): position 1936 A/G SNP.	Increase breast and colorectal cancer, increased heart rate and risk of myocardial infarction	Decreased risk of preterm birth
Angiotensin-Converting Enzyme (ACE): 287 bp Insertion/deletion	Increased risk hypertension, coronary artery disease, myocardial infarction, polycystic ovary syndrome, prostate cancer	Improved acute cardiovascular fitness, decreased risk of Alzheimer’s disease and migraines, protective vs. SARS-CoV-2
Apolipoprotein E (ApoE): E4, E3, and E2 alleles	Increased risk hypertension, cardiovascular disease, Alzheimer’s disease	Increased fertility, cognitive function, and resistance to liver disease; reduced risk from severe childhood diarrhea
Breast Cancer Type 1 (BRCA1): position 185 AG deletion	Increased risk of breast, ovarian, endometrial, and prostate cancer	Increased reproduction
Breast Cancer Type 2 (BRCA2): position 6174 T deletion	Increased risk of melanoma and breast, ovarian, and pancreatic cancers	Increased reproduction
Cystic Fibrosis (CFTR): position 508 Phe deletion	Obstructive lung disease, respiratory infection, pancreatic disorders, reduced fertility	Resistance to tuberculosis (and maybe cholera and SARS-CoV-2)
Hemochromatosis (HFE): position 282 Cys/Tyr polymorphism	Chronic fatigue, hyperpigmentation, joint and bone disorders, liver disorders	Improved general fitness, reduced risk of amyotrophic lateral sclerosis, Alzheimer’s disease, Parkinsons disease, atherosclerosis; resistance to tuberculosis
Human Leukocyte Antigen (HLA): 8.1 ancestral haplotype (AH)	Increased risk of non-Hodgkin lymphoma, colorectal and ovarian cancer, diabetes, autoimmune disorders, and myositis	Increase male longevity, resistance to *Staphylococcus aureus* and *Pseudomonas aeruginosa* and septic shock in cystic fibrosis (CF) patients, protective *versus* narcolepsy
Machado-Joseph Disease (MJD): CAG trinucleotide repeat number	Progressive clumsiness, lurching gait, impaired eye movement, and dystonia	Increase reproduction
Methylenetetrahydrofolate Reductase (MTHFR): Position 677 C/T SNP	Increased risk of birth defects, schizophrenia, infertility, gestational diabetes, cervical cancer, recurrent pregnancy loss, reduced fertility, polycystic ovary syndrome, and Alzheimer’s disease	Reduced risk of colorectal cancer
Niemann-Pick Disease, type C1 (NPC1): many alleles	Liver, spleen, brain, and motor control problems; death before adulthood	Resistance to filoviruses (Ebola and Marburg) - evidence from tissue culture and mouse studies
R*hesus* (RH) factor blood groups: positive and negative for D locus	General poor health, fetal anemia and mother’s immune system attacks fetus	Increase reproduction, reduced pregnancy induced hypertension, resistance to Chikungunya fever; frequency dependent mechanism

**TABLE 2:** Antagonistically pleiotropic disease alleles described in three other review papers (Carter and Nguyen, 2011; Withrock et al., 2015; Ukraintseva et al., 2016; Byars and Voskarides, 2020) and this one. For the 39 loci/alleles listed, the name and the review papers in which they are described is shown. Descriptions that include studies using human, epidemiological or clinical data are shown with “XXX” symbols while descriptions that rely exclusively on animal models or circumstantial evidence are shown with the “X" symbol.

Disease or locus name	Carter and Nguyen (2011)	Withrock et al., 2015	Ukraintseva et al. (2016)	Byars and Voskarides (2020)	This paper
A kinase anchor protein 2 (AKAP10)					XXX
Angiotensin I converting enzyme (ACE)			X		XXX
Androgen receptor (AR)	XXX				
Apolipoprotein B variable number of tandem repeats region 3'(3′-APOB-VNTR)			X		
Apolipoprotein E e4 (ApoE e4)			X		XXX
Beta-thallasemia	XXX	XXX			
Breast cancer 1 (BRCA1)				XXX	XXX
Breast cancer 2 (BRCA2)				XXX	XXX
C-C chemokine receptor type 5 (CCR5)				XXX	
CDG-11b (MOGS)		X			
Coronary heart disease				XXX	
Cystic fibrosis (CF)	XXX	XXX		XXX	XXX
Cytotoxic T lymphocyte antigen-4 (CTLA-4)			X		
Duffy antigen		X			
Enoyl Coenzyme A hydratase, short chain, 1(ECHS1)			X		
Epithelial cancer (TNFRSF11B)	X				
Glucose-6-phosphate dehydrogenase deficiency (G6PD)	XXX	XXX			
Hemochromatosis (HFE)	X			XXX	XXX
Hemosiderosis		X			
Hepatocellular carcinoma (PTNP11)	X				
Human Leukocyte antigen (HLA)					XXX
Huntington’s Disease (HTT)	XXX			XXX	
Lipoprotein Lp(a) (LPA)			X		
Machado-Jacob disease (MJD)					XXX
Methylenetetrahydrofolate reductase (MTHFR)			X		XXX
Myasthenia gravis (MG)		X			
Niemann-Pick disease, type C1 (NPC1)		X			X
NRDE2			X		
Osteoporosis (ALOX15)	XXX				
Phenylketoneurea (PKU)	X	XXX	X		
Plasminogen activator inhibitor-1 (PAI-1)			X		
Pyruvate kinase deficiency		XXX			
RH blood groups (RH)					XXX
Schizophrenia (6p22-p24, 11q21-22)	X				
Sickle cell (Hbb)	XXX	XXX			
Superoxide dismutase 2 (SOD2)			X		
Tay-Sachs (HEXA)	X	XXX			
Triosephosphate isomerase deficiency (TPI)	X				
Tumor protein p53 (TP53)			X		

The number of examples identified is likely to be an underestimate due to the relative lack of attention given to identifying and demonstrating beneficial effects in genetic disease research. The examples we presented relied on focused studies examining the individual mechanisms by which fitness or medical differences are evident. These well-supported examples of antagonistic pleiotropy therefore come from studies of individual loci of pre-existing interest for which the negative effects have been well-studied and often the beneficial effects were discovered incidentally. The number of individual loci that have received this degree of attention, from a genome with over 20,000 loci, represents a small minority of possible candidates.

Because of the historical attention paid to antagonistic pleiotropy (e.g., sickle cell anemia), and the relatively straightforward way to identify it (i.e., identify benefits and detriments as done above), it represents a form of balancing selection familiar to many individuals interested in medical genetics. Balancing selection encompasses a wider range of phenomena than just antagonistic pleiotropy however.

The theoretical importance of balancing selection, selection which acts to maintain multiple alleles and genetic diversity rather than selection which purges diversity, and a few key examples have been known for a long time (Fisher, 1922; Dobzhansky, 1955; Lewontin, 1987). Several different mechanisms which lead to balancing selection have been identified, each with their own distinct causes and dynamics.

The first mechanism, heterozygote advantage or heterosis, was first described in Fisher’s foundational paper (Fisher, 1922; Charlesworth, 2022) where he argued for a process in which higher fitness in heterozygotes than in either homozygote could act to preserve genetic diversity at a locus. This scenario is best exemplified by the classic sickle cell example in which heterozygotes for the sickle cell mutation experience improved resistance to malaria while rarely suffering the negative effects of blood cell sickling (Esoh and Wonkam, 2021). This mechanism is most likely to be present when the deleterious effects of an otherwise beneficial mutation tend to be recessive.

Another cause of heterosis is situations in which increased variation at a locus is inherently advantageous, with neither allele at a locus truly inferior to the other but the presence of distinct alleles providing a benefit. Such a process appears to be important for loci which code for olfactory receptors (Alonso et al., 2008) and immune system recognition proteins like the MHC (Radwan et al., 2020) and Toll-like receptors (TLRs) (Minias and Vikler, 2022), proteins involved in biological processes in which a wider array of distinct proteins is advantageous.

A second balancing selection mechanism is negative frequency dependent selection, a process in which an allele’s fitness increases as it becomes rarer (Brisson, 2018; Christie and McNickle, 2023). In this manner multiple alleles can be maintained because whenever one becomes rare it experiences increased fitness, driving the other allele lower in frequency which then benefits that second allele, creating a cycle which maintains both alleles. While often studied in the context of behavioral processes and sexual selection where individuals with the unusual behavior or phenotype experiences higher fitness (Janif et al., 2014; Uhl and Carter, 2023), recent studies have also examined evidence for negative frequency dependence in medically relevant human alleles (Villanea et al., 2015). As an example of this process, in our discussion of the Rh blood group factors above, the reduction in fitness cost to females with the Rh negative allele when it is rare is a major aspect of what allows it to persist. Despite its potential for influencing human health, studies of this mechanism appear underrepresented in the medical literature.

A third mechanism which leads to balancing selection is sexual conflict. This describes a situation in which an allele is advantageous in one sex while deleterious in the other (Bonduriansky and Chenoweth, 2009; Kasimatis et al., 2017). This arises most naturally for traits related to sexual reproduction and primary sex characteristics, but can apply to any trait for which the sexes differ. Intralocus sexual conflict describes alleles at the same locus having opposing fitness effects in the sexes whereas interlocus sexual conflict describes similar interactions across multiple loci. Of these, intralocus sexual conflict is more directly tied to balancing selection. As an example, men and women differ in optimal height and alleles at loci influencing height are selected in different directions (Stulp et al., 2012). Also, as described above, the 8.1 AH allele in the HLA complex appears more beneficial in males while more deleterious in females (Caruso et al., 2000). Genetic studies of specific loci involved in sexual conflict with detailed fitness measurements are rare and come mainly from animal studies (Barson et al., 2015).

A fourth mechanism of balancing selection comes from antagonistic pleiotropy. As described in the introduction, the initial conceptions of antagonistic pleiotropy were strongly associated with aging (Medawar, 1951; Williams, 1957; Kirkwood and Rose, 1991), one function being good in youth at the expense of another being worse at older age. Several of the examples discussed above (e.g., AKAP, ApoE, BRCA1/2, MJD) can be seen in this light, but the majority of our examples do not fall cleanly and entirely within this classic senescence-based viewpoint. A recent study examining genetic, reproductive, and lifespan data for over 275,000 individuals showed results consistent with the presence of this senescence-based antagonistic pleiotropy (Long and Zhang, 2023), but the presence of this type of antagonistic pleiotropy does not undermine arguments for the prevalence of other types of antagonistic pleiotropy.

In this paper we have used a broader concept of antagonistic pleiotropy to describe any locus in which alleles can have multiple effects on fitness, whether at different stages of life, across different sexes, or in different physiological systems within the same individual (Bitarello et al., 2023). Our focus has been on alleles that would otherwise be classified as named diseases or serve as major risk factors for health conditions.

A fifth mechanism of balancing selection is selection driven by spatial variation in optimal alleles across a reproductively connected population. In this scenario, different alleles may be positively selected in different regions or subpopulations of the population and result in the persistence of multiple alleles. Alleles that are beneficial in one region may be detrimental and even be classified as “diseases” in another. The classic sickle cell example exhibits aspects of this mechanism, whereby the heterozygote advantage possessed in regions with malaria is absent in regions without, which accounts for its geographic distribution (Piel et al., 2010). As described above, cystic fibrosis shows geographic patterns consistent with the spatial distribution of tuberculosis (Lubinsky, 2012; Bosch et al., 2017).

A sixth mechanism of balancing selection is driven by temporal variation, termed fluctuating selection. Fluctuating selection may be involved in the maintenance of some immune system alleles since diseases vary over time and the optimal alleles in immune system pathogen recognition systems are therefore unlikely to remain the same. The analysis of TLRs cited above as an example of heterosis reported that while both processes were likely important, the evidence for fluctuating selection as the cause of maintained variation was stronger (Minias and Vikler, 2022).

These six mechanisms of balancing selection are not mutually exclusive and individual genetic diseases may experience several of these simultaneously. Examples of antagonistic pleiotropy as we have considered in this paper often bridge the divides between these different balancing selection mechanisms. The cystic fibrosis example described above combines elements of antagonistic pleiotropy (i.e., lung illness and disease resistance and an illness) with spatial selection (i.e., geographic patterns of disease prevalence) and fluctuating selection (i.e., historical periods of high and low rates of disease).

Studies which identify the details of the multiple selective forces at play in the variety of types of balancing selection described above are relatively labor intensive, with multiple studies needed to elucidate the details for each example. Other methods which rely on broader large-scale statistical analyses may also reveal evidence of antagonistic pleiotropy. More precisely, these studies look for genomic evidence of balancing selection.

Building on older statistical tests for selection in individual alleles (Tajima, 1989; Kreitman, 2000), genomic data can be analyzed for signs of selection in alleles (Key et al., 2014; Fijarczyk and Babik, 2015; Quintana-Murci, 2016; Bitarello et al., 2018).

Genomic studies have revealed evidence for patterns of selection consistent with antagonistic pleiotropy or other forms of balancing selection, but often the details are less than complete. For example, an analysis of an allele at the cadherin related family member 3 (CHDR3) receptor locus showed evidence of selection more consistent with negative frequency-dependent selection than the expected positive selection, but the authors were unclear on what the cause of that selection may be (O'Neill et al., 2020). Similarly, the human salivary agglutinin gene (DMBT1) gene shows population genetic evidence for balancing selection and one allele is known to inhibit infection by *Streptococcus mutans*, but the other selection factors that result in balancing selection instead of purely positive selection are unknown (Alharbi et al., 2022). In cases such as these, the story is incomplete - with statistical evidence for multiple opposing selective forces at play and one factor identified, but the opposing factor unknown.

In fact, multiple genomic studies now provide evidence for balancing selection at large numbers of loci but are then left to speculate on the selective factors in the absence of direct studies of the individual alleles. Typically, these are hypothesis-free studies looking for genetic patterns of balancing selection and identifying them in a large number of loci, but often without any more detail than knowing the functional classes of the loci (e.g., Andrés et al., 2009; Abraham et al., 2022; Velazquez-Arcelay et al., 2022; Aqil et al., 2023). While reliable and complete identification of the types of balancing selection revealed are unresolved, at a minimum it seems that previous suggestions that balancing selection is rare or irrelevant (Asthana et al., 2005; Hedrick, 2012) are weakened by this increasing evidence.

Recently, some genomic studies have explicitly looked for antagonistic pleiotropy. For example, a GWAS approach revealed many loci which have been associated with autism spectrum disorders also showed evidence of positive selection, but those antagonistic positive effects are unknown (Polimanti and Gelernter, 2017). An interesting broader recent approach has been to attempt a direct test of predictions from the traditional, senescence-based, antagonistic pleiotropy model whereby deleterious alleles which effect the young should be rarer than those that effect the old (Rodríguez et al., 2017; Long and Zhang, 2019; Rodríguez et al., 2019). This general pattern was observed, and about 10% of these early-effect alleles showed evidence of some kind of pleiotropy (Rodríguez et al., 2017). A related study on fixation rates of early and late expressed genes showed similar patterns of stronger purifying selection on early vs. late expressed genes (Cheng and Kirkpatrick, 2021). These methods cannot cleanly distinguish between alleles differentially selected due to antagonistic pleiotropy or mutation accumulation (a non-pleiotropy model for senescence which predicts a similar pattern) however, so more studies like this should be done.

If antagonistic pleiotropy is as widespread as we suggest it may be, the implications for humanity’s long-term evolutionary future may be profound. With many deleterious effects of genetic disease reduced by modern medicine, the continued benefits conferred by these alleles would contribute to a higher net advantage and these disease alleles may therefore be expected to increase in frequency from their current levels, making genetic disease more common over time. This process will be a slow one however, taking many generations to make meaningful changes in the population.

In the short-term, the prevalent nature of antagonistic pleiotropy presents two major implications for the research and treatment of genetic diseases.

First, identification of pleiotropic alleles that confer protection from diseases and understanding their mechanisms of action may aid medical and epidemiological research. Novel treatments for certain conditions may arise from studying these alleles. For example, by understanding the mechanisms utilized by mutations in the BRCA1 and BRCA2 to increase reproductive fitness, we may be able to further our understanding of the reproductive system and potentially discover novel treatments for infertility.

Second, our results also suggest that genetically based treatments for diseases must be used with caution. The CRISPR/Cas9 gene editing technique has potential to treat genetic diseases (Hsu et al., 2014; Sander and Joung, 2014; Abdelnour et al., 2021). At present, primary concerns in the development of these techniques include off-site modifications in addition to the intended ones (Liang et al., 2015; Wang and Wang, 2019) or *de novo* LINE-1 insertions (Tao et al., 2022), but even if specificity of the modification is perfect such genetic techniques should be used carefully to avoid negative consequences arising from the loss of a beneficial pleiotropic effect.

In cases where benefits from a genetic disease allele do not exist, or when the disease allele is extremely deleterious or lethal, complete editing of the disease allele to the wildtype may be the best approach. However, for many diseases with smaller effects the calculation is different. The lack of attention paid to potential pleiotropic benefits means that widespread genome editing may inadvertently eliminate many presently unrecognized yet beneficial genetic effects in our population. For example, using gene editing techniques to correct mutations in the AKAP10 allele may decrease risk for breast cancer and colorectal cancer, but at the cost of increasing the risk of pre-term birth. Recognizing this duality of effects may allow us to recommend treatment schedules such as one in which an AKAP10-focused genetic treatment to reduce cancer risk be delayed until after child-bearing years to avoid the increased risk of pre-term births.

Ideally, instead of completely editing or silencing a disease allele, selectively editing the deleterious aspects of the pleiotropic system while still maintaining the beneficial ones provides a more optimal approach. The first step in looking for these pleiotropic effects and creating these better treatments is an appreciation for the potential ubiquity of antagonistic pleiotropy.
